# Muscle-specific TGR5 overexpression improves glucose clearance in glucose-intolerant mice

**DOI:** 10.1074/jbc.RA120.016203

**Published:** 2020-12-04

**Authors:** Takashi Sasaki, Yuichi Watanabe, Ayane Kuboyama, Akira Oikawa, Makoto Shimizu, Yoshio Yamauchi, Ryuichiro Sato

**Affiliations:** 1 Food Biochemistry Laboratory, Department of Applied Biological Chemistry, Graduate School of Agricultural and Life Sciences, University of Tokyo, Tokyo, Japan; 2 RIKEN Center for Sustainable Resource Science, Yokohama, Kanagawa, Japan; 3 Faculty of Agriculture, Yamagata University, Tsuruoka, Yamagata, Japan; 4 Nutri-Life Science Laboratory, Department of Applied Biological Chemistry, Graduate School of Agricultural and Life Sciences, University of Tokyo, Tokyo, Japan; 5 AMED-CREST, Japan Agency for Medical Research and Development, Tokyo, Japan

**Keywords:** skeletal muscle metabolism, energy metabolism, muscle hypertrophy, G protein–coupled receptor, bile acid, obesity, aging, diabetes, BAT, brown adipose tissue, CE-TOF MS, capillary electrophoresis time-of-flight mass spectrometry, F6P, fructose 6-phosphate, G6P, glucose 6-phosphate, HFD, high-fat diet, IPGTT, intraperitoneal glucose tolerance test, ITT, insulin tolerance test, ND, normal diet, NEFA, nonesterified fatty acid, OGTT, oral glucose tolerance test, PFK, phosphofructokinase, RER, respiratory exchange ratio, TG, triacylglycerol, TLCA, taurolithocholic acid, WAT, white adipose tissue

## Abstract

TGR5, a G protein–coupled bile acid receptor, is expressed in various tissues and regulates several physiological processes. In the skeletal muscle, TGR5 activation is known to induce muscle hypertrophy; however, the effects on glucose and lipid metabolism are not well understood, despite the fact that the skeletal muscle plays a major role in energy metabolism. Here, we demonstrate that skeletal muscle–specific TGR5 transgenic (Tg) mice exhibit increased glucose utilization, without altering the expression of major genes related to glucose and lipid metabolism. Metabolite profiling analysis by capillary electrophoresis time-of-flight mass spectrometry showed that glycolytic flux was activated in the skeletal muscle of Tg mice, leading to an increase in glucose utilization. Upon long-term, high-fat diet challenge, blood glucose clearance was improved in Tg mice without an accompanying increase in insulin sensitivity in skeletal muscle and a reduction of body weight. Moreover, Tg mice showed improved age-associated glucose intolerance. These results strongly suggest that TGR5 ameliorated glucose metabolism disorder that is caused by diet-induced obesity and aging by enhancing the glucose metabolic capacity of the skeletal muscle. Our study demonstrates that TGR5 activation in the skeletal muscle is effective in improving glucose metabolism and may be beneficial in developing a novel strategy for the prevention or treatment of hyperglycemia.

Bile acids, the primary component of bile, are released from the gallbladder after meals to promote the absorption of lipids and fat-soluble vitamins in the small intestine. Almost 95% of bile acids are reabsorbed in the ileum and transported back to the liver through the portal blood and recycled. Therefore, the concentration of blood bile acids temporarily reaches high levels in the postprandial state (1). Interestingly, blood bile acids have been reported to function as metabolic regulators by activating several bile acid receptors.

TGR5 (also known as G protein–coupled bile acid receptor 1) is a G protein–coupled receptor that exists in the plasma membrane and recognizes bile acid as its ligand (2, 3). Ligand-bound TGR5 interacts with Gαs subunit and then activates the cAMP signaling pathway. TGR5 is expressed in various tissues, such as brown adipose tissue (BAT), white adipose tissue (WAT), and intestinal L cells. In BAT and WAT, TGR5 promotes energy expenditure, causing the amelioration of obesity (4, 5, 6). In contrast, TGR5 activation in intestinal L cells enhances GLP-1 secretion and improves diabetes in mice (7, 8). TGR5 is also expressed in the skeletal muscle, and its expression is increased by exercise (9). We have previously demonstrated that TGR5 activation induces muscle cell differentiation in cultured muscle cells and muscle hypertrophy in mice (9). Because skeletal muscle–specific TGR5 overexpression increases muscle strength, TGR5 may be a feasible target for maintaining muscle function. In fact, several compounds possessing TGR5 agonistic activity, such as citrus limonoid nomilin and obacunone, have antiobesity, antidiabetic, and muscle hypertrophy effects (10, 11, 12).

As the skeletal muscle is not only central to the locomotor system but also the largest glucose-metabolizing organ, a higher muscle mass is associated with better glycemic control (13). However, a lower skeletal muscle mass is significantly associated with type 2 diabetes (14), suggesting that increasing the skeletal muscle mass is effective in improving diabetes. Therefore, it could be expected that TGR5 activation in the skeletal muscle improves glucose metabolism by inducing muscle hypertrophy, although this has not been verified till date.

In this study, we evaluated the effects of muscle TGR5 on energy metabolism using skeletal muscle–specific hTGR5 Tg mice. Interestingly, skeletal muscle–specific overexpression of TGR5 induced an increase in the respiratory exchange ratio (RER) along with the activation of glycolytic flux in the skeletal muscle. As anticipated from these results and the fact that TGR5 induces muscle hypertrophy, Tg mice exhibited better glucose clearance under long-term high-fat diet (HFD) challenge, which was independent of changes in muscle insulin sensitivity. We also observed that Tg mice showed improvement in aging-associated glucose intolerance. Altogether, our study indicates that muscle TGR5 activation contributes to improving glucose intolerance by increasing muscle mass and glucose utilization and may be beneficial in developing a novel strategy for the prevention or treatment of hyperglycemia caused by obesity and aging.

## Results

### Muscle-specific TGR5 overexpression increased RER and glycolytic flux without changing the gene expression related to glucose and lipid metabolism in mice

To evaluate the effect of muscle TGR5 on energy metabolism, we compared muscle weight and the expression of several genes related to glucose and lipid metabolism between WT and Tg mice. As we had reported previously, Tg mice exhibited significantly increased muscle weight (Fig. S1*A*); however, no differences were observed in mRNA expression involved in lipid and glucose metabolism (Fig. S1*B*). Moreover, periodic acid–Schiff staining of gastrocnemius muscle samples revealed almost identical glycogen storage between WT and Tg mice (Fig. S1*C*).

**Figure 1:**
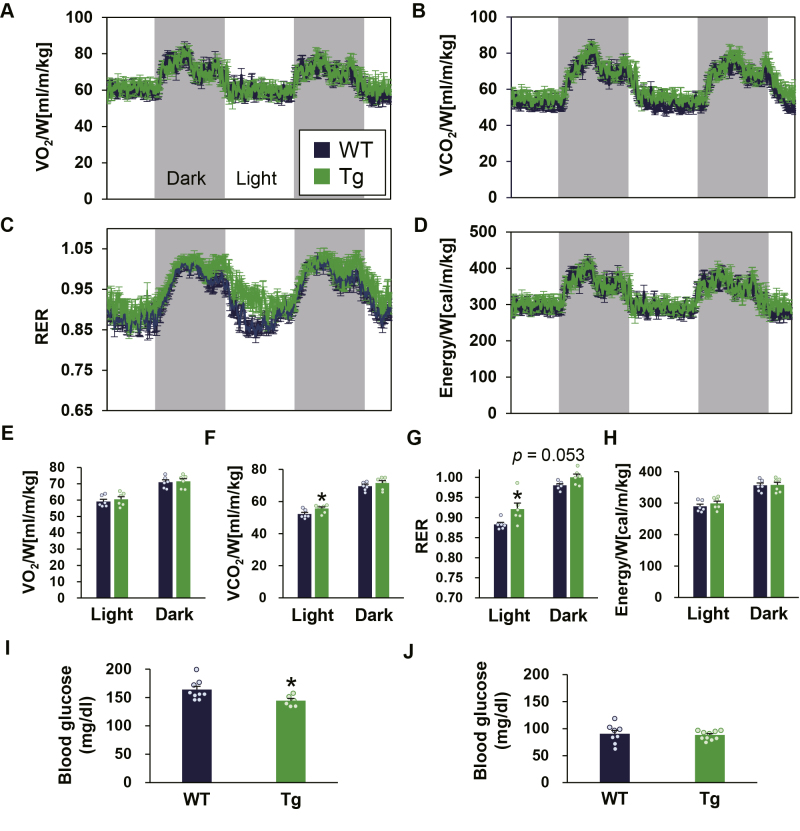
**Tg mice exhibit significantly higher RER under a normal diet.***A–D*, RER and energy expenditure, evaluated by oxygen consumption and carbon dioxide production, were monitored for 48 h under normal diet (n = 6). *E–H*, average values in light and dark periods (n = 6). *I*, blood glucose levels in the free-feeding condition (n = 6–9). *J*, blood glucose levels after fasting for 18 h. Data are mean ± SE. Statistical analyses were conducted using a two-tailed unpaired Student’s *t* test. ∗, *p* < 0.05. RER, respiratory exchange ratio.

We next measured the RER and energy expenditure, which was calculated from O_2_ consumption (VO_2_) and CO_2_ production (VCO_2_), in Tg and WT mice fed a normal diet (ND) (Fig. 1, *A*–*H*). Although no difference was detected in VO_2_ and energy expenditure in both light and dark periods between the two groups (Fig. 1, *A* and *D*, E, H), VCO_2_ and RER were significantly higher in Tg mice in the light period and showed an increasing trend in the dark period (*p* = 0.053) (Fig. 1, *B, C, F* and *G*), thus indicating the preferential use of carbohydrates in Tg mice compared with WT mice. Consistent with these results, the blood glucose levels of Tg mice were lower than those of control littermates in the free-feeding condition, but were comparable in the fasting condition (Fig. 1, *I* and *J*).

**Figure 2:**
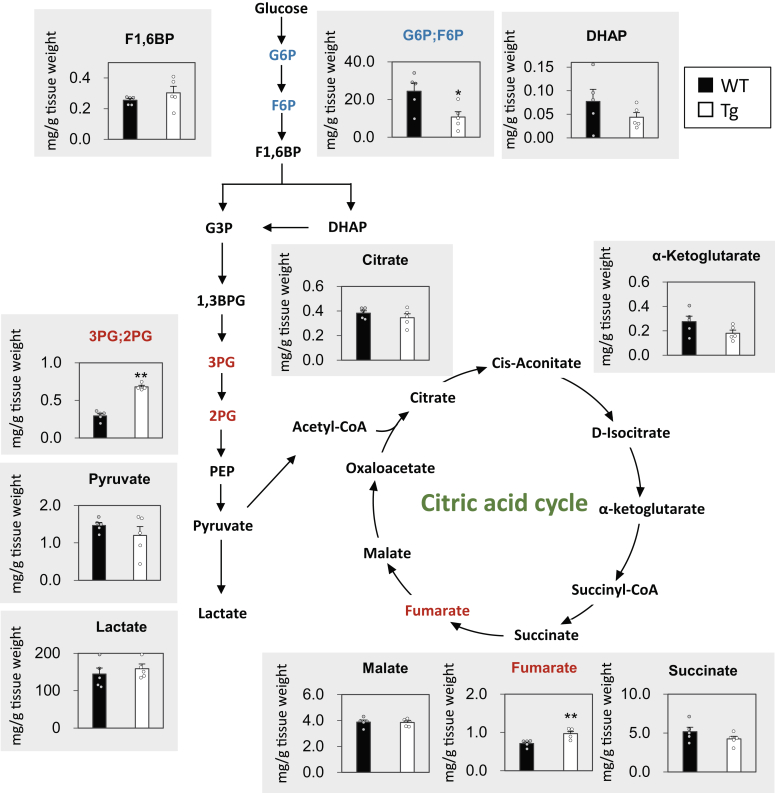
**TGR5 activates glycolytic flux in skeletal muscle.** Comparison of major metabolites produced in glycolysis and citric acid cycle in gastrocnemius muscle (n = 5). *Red* indicates a significant increase and *blue* indicates a significant decrease in Tg mice. Data are mean ± SE. Statistical analyses were conducted using a two-tailed unpaired Student’s *t* test. ∗, *p* < 0.05; ∗∗, *p* < 0.01. 1,3BPG, 1,3-Bisphosphoglycerate; 2PG, 2-phosphoglycerate; 3PG, 3-phosphoglycerate; DHAP, dihydroxyacetone phosphate; F1,6BP, fructose-1,6-bisphosphate; F6P, fructose 6-phosphate; G3P, glyceraldehyde 3-phosphate; G6P, glucose 6-phosphate; PEP, phosphoenolpyruvic acid.

To evaluate whether TGR5 affects glycolytic flux, we performed capillary electrophoresis time-of-flight mass spectrometry (CE-TOF MS) analysis of major metabolites produced in the glycolysis and citric acid cycle in the skeletal muscle (Fig. 2). For the glycolytic metabolite, the levels of glucose 6-phosphate (G6P) and fructose 6-phosphate (F6P) were significantly reduced (0.44-fold) in Tg mice. In particular, F6P was a substrate for phosphofructokinase (PFK), the most important rate-limiting enzyme in glycolysis. No significant difference was observed between WT and Tg mice for several metabolites such as fructose-1,6-bisphosphate (F1,6BP), dihydroxyacetone phosphate, pyruvate, and lactate; however, the levels of 3-phosphoglycerate (3PG) and 2-phosphoglycerate (2PG) were 2.3-fold higher in Tg mice. These results indicate the activation of glycolytic flux in TGR5-overexpressed skeletal muscle. On the other hand, for the metabolites of the citric acid cycle, no significant change was detected in the levels of citrate, α-ketoglutarate, succinate, and malate. Although a slight increase (1.37-fold) was observed in fumaric acid levels, the effect of TGR5 on the citric acid cycle was found to be minimal (Fig. 2).

### Muscle TGR5 did not affect lipid metabolism

**Figure 3:**
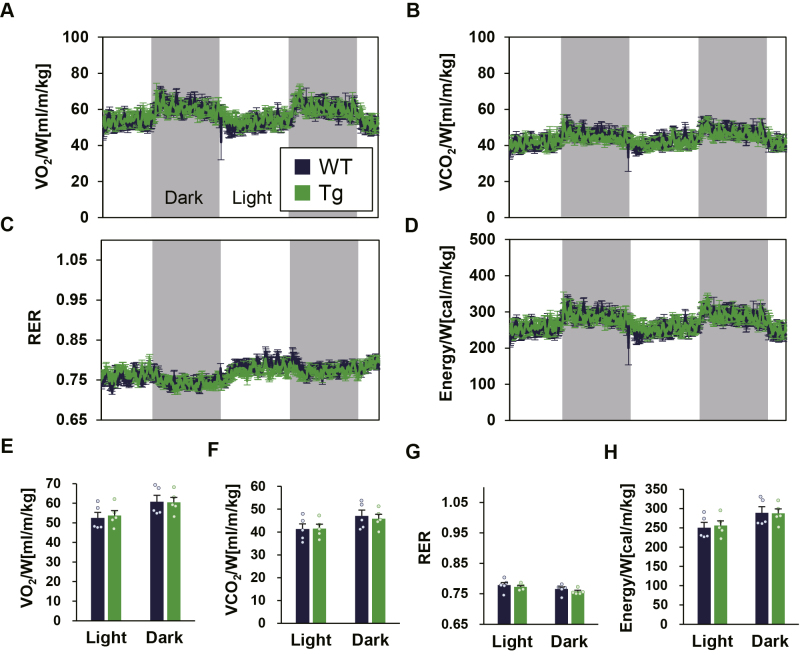
**WT and Tg mice show similar RER under HFD.***A–D*, RER and energy expenditure, evaluated by oxygen consumption and carbon dioxide production, were monitored for 48 h under 4 weeks of HFD. *E–H*, the average value in light and dark periods (n = 5). Data are mean ± S.E. Statistical analyses were conducted using a two-tailed unpaired Student’s *t* test. HFD, high-fat diet; RER, respiratory exchange ratio.

To understand whether muscle TGR5 affects lipid metabolism, we conducted the expired gas analysis again using HFD-challenged mice and calculated the RER and energy expenditure from VO_2_ and VCO_2_ (Fig. 3, *A*–*H*). The average RER was <0.8, indicating that most of the energy is fat-dependent (Fig. 3, *C* and *G*). As observed in the case of ND, there was no difference in energy expenditure in both light and dark periods between WT and Tg mice (Fig. 3, *D* and *H*). However, there was also no difference in the RER that was significantly higher in Tg mice under ND. These results indicate that the increase in glucose utilization ratio by skeletal muscle TGR5 is induced only when there is sufficient glucose available as an energy source and that muscle TGR5 has little effect on lipid metabolism.

**Figure 4:**
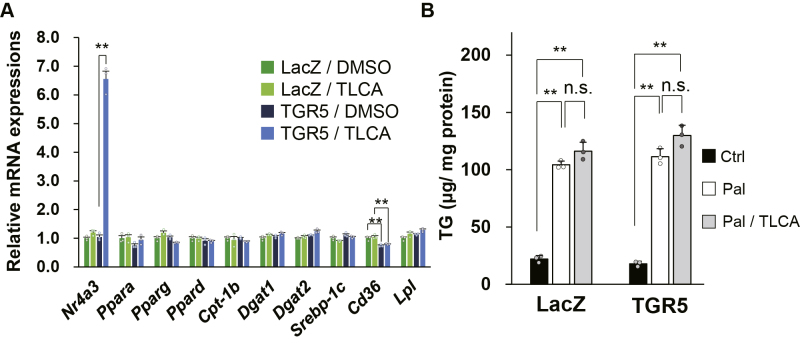
**TGR5 activation does not affect lipid metabolism.***A*, C2C12 myotubes infected with adenovirus expressing TGR5 or LacZ were treated with TLCA (50 μM) for 3 h. mRNA levels were determined by RT-PCR (n = 3). *B*, TG accumulation normalized by total protein in C2C12 myotubes expressing LacZ or TGR5 and treated with palmitate (250 μM) and TLCA (50 μM) for 18 h (n = 3). Data are mean ± S.E. Statistical analyses were conducted using one-way ANOVA (Tukey–Kramer post hoc test). ∗, *p* < 0.05; ∗∗, *p* < 0.01; n.s., not significant. TG, triacylglycerol; TLCA, taurolithocholic acid.

To confirm that TGR5 seldom affects lipid metabolism in myocytes, C2C12 myotubes expressing TGR5 by adenovirus were treated with taurolithocholic acid (TLCA), one of the most potent endogenous TGR5 ligands, and the expression of genes involved in lipid metabolism was measured (Fig. 4*A*). Acute TGR5 activation in C2C12 myotubes increased the mRNA level of Nr4a3 that was selectively and profoundly upregulated by PKA in response to TGR5 activation (9). TGR5-expressing C2C12 myotubes exhibited a slightly lower expression of Cd36, but the response appeared to be independent of TGR5 activation because the expression level was not altered by stimulation with TGR5 ligand. The expression of other major lipid metabolism genes remained unaffected by TGR5 activation (Fig. 4*A*). Next, LacZ- or TGR5-expressing C2C12 myotubes were incubated with palmitate and TLCA for 18 h to explore the effect of TGR5 on intracellular triacylglycerol (TG) accumulation. As anticipated from our data indicating that muscle TGR5 activation does not alter energy expenditure and gene expressions involved in lipid metabolism (Fig. 3 and 4*A*), intracellular TG accumulation was comparable between TLCA-treated and TLCA-untreated C2C12 myotubes, despite the overexpression of TGR5 (Fig. 4*B*).

### Tg mice exhibited improved glucose clearance under HFD challenge

**Figure 5:**
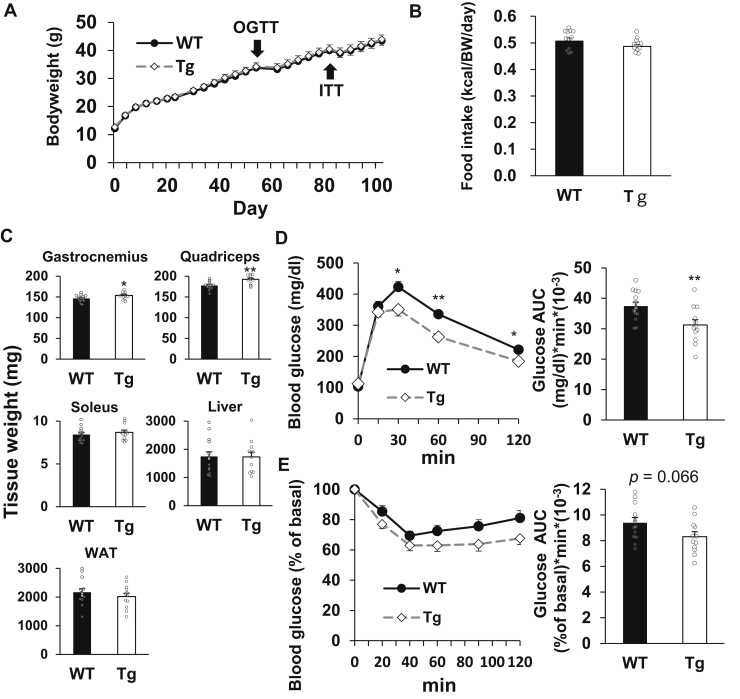
**Muscle TGR5 preserves obesity-induced dysfunction of glucose homeostasis.***A–C*, body weight (*A*), food intake (*B*), and tissue weight (*C*) of WT mice and Tg littermates fed HFD. *D, E*, oral glucose tolerance test (*D*) and insulin tolerance test (*E*). The *right panel* shows AUC. Data are mean ± S.E. (n = 12–14). Statistical analyses were conducted using a two-tailed unpaired Student’s *t* test. ∗, *p* < 0.05; ∗∗, *p* < 0.01. AUC, area under the curve; HFD, high-fat diet; OGTT, oral glucose tolerance test; ITT, insulin tolerance test.

Based on our results indicating that TGR5 induces muscle hypertrophy and increases glucose utilization, we next evaluated the effect of TGR5 on glucose clearance. Since the oral glucose tolerance test (OGTT) did not show a significant difference between WT and Tg mice fed ND (Fig. S2), we performed a series of experiences using HFD-induced hyperglycemic mice. During 15 weeks of HFD challenge, we observed no significant differences in body weight and food intake between Tg and WT littermate mice (Fig. 5, *A* and *B*). These results were consistent with the unchanged energy expenditure between Tg and WT mice (Fig. 3, *D* and *H*). There were significant increases in the weights of gastrocnemius and quadriceps muscles (5.0% and 8.5%, respectively), despite the lack of changes in the body weight of Tg mice (Fig. 5, *A* and *C*). In contrast, the weights of the soleus muscle, liver, and WAT were comparable between Tg and WT mice (Fig. 5*C*). Interestingly, the OGTT demonstrated improved glucose clearance in Tg mice challenged with HFD for 8 weeks compared with WT mice (Fig. 5*D*). Moreover, the insulin tolerance test (ITT) disclosed that Tg mice fed HFD for 12 weeks had slightly improved glucose clearance compared with WT mice (Fig. 5*E*). Although the levels of fasting plasma glucose, TG, nonesterified fatty acids (NEFAs), and insulin, which are indicators of insulin resistance, showed no differences between Tg and WT mice (Fig. S3), our data suggest the importance of muscle TGR5 in preventing obesity-induced dysfunction of glucose metabolism. Similar results were also obtained from another transgenic line, which was described in our previous report (9). Briefly, skeletal muscle TGR5 did not prevent HFD-induced obesity and did not alter liver and WAT weight; however, it did significantly increase the skeletal muscle weight. Moreover, Tg mice exhibited improved blood glucose clearance as evaluated by the OGTT and ITT (Fig. S4).

### TGR5 did not improve insulin resistance in muscle cells

**Figure 6:**
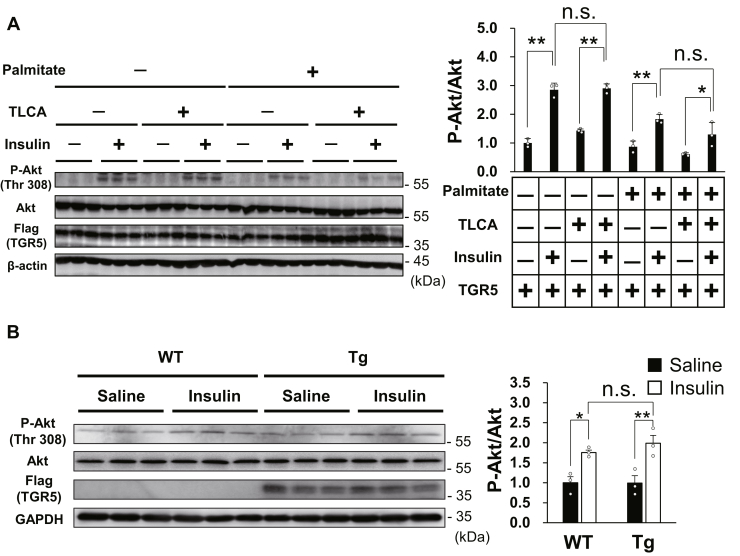
**Muscle TGR5 does not improve insulin sensitivity.***A, B*, Akt phospho- and total protein, FLAG, and β-actin or GAPDH protein were measured by Western blotting. *A*, C2C12 myotubes were precultured with palmitate (500 μM) and TLCA (50 μM) for 18 h and then treated with insulin (100 nM) for 30 min (B) WT and Tg littermates fed an HFD for 8 weeks were injected with saline or insulin (0.75 IU/kg body weight) for 30 min after 18 h of fasting and then the gastrocnemius muscle was isolated. Data are mean ± S.E. Statistical analyses were conducted using one-way ANOVA (Tukey–Kramer post hoc test). ∗, *p* < 0.05; ∗∗, *p* < 0.01; n.s., not significant. HFD, high-fat diet; TLCA, taurolithocholic acid.

Considering that muscle TGR5 can improve glucose clearance in mice with HFD-induced obesity, we hypothesized that TGR5 activation would ameliorate insulin resistance. To assess whether short-term TGR5 activation improves insulin resistance in muscle cells, we evaluated insulin-induced Akt phosphorylation of TGR5-expressing C2C12 myotubes that developed insulin resistance by palmitate. As anticipated, insulin stimulation immediately increased Akt phosphorylation, which was attenuated by palmitate pretreatment (Fig. 6*A*). In contrast, TGR5 activation by TLCA did not alter insulin sensitivity in both palmitate-treated and palmitate-untreated C2C12 myotubes (Fig. 6*A*). We next evaluated the effects of long-term muscle TGR5 activation on insulin sensitivity using WT and Tg littermates, which were fed HFD for 8 weeks. In response to insulin injection, Akt was phosphorylated within 30 min in the skeletal muscle of both WT and Tg mice, but similar to the short-term *in vitro* experience, muscle TGR5 activation had no influence on muscle insulin sensitivity (Fig. 6*B*). These results indicate that TGR5 activation in the skeletal muscle has no beneficial effect on the improvement of insulin resistance due to lipotoxicity.

### Skeletal muscle TGR5 improved glucose clearance in elderly mice

**Figure 7:**
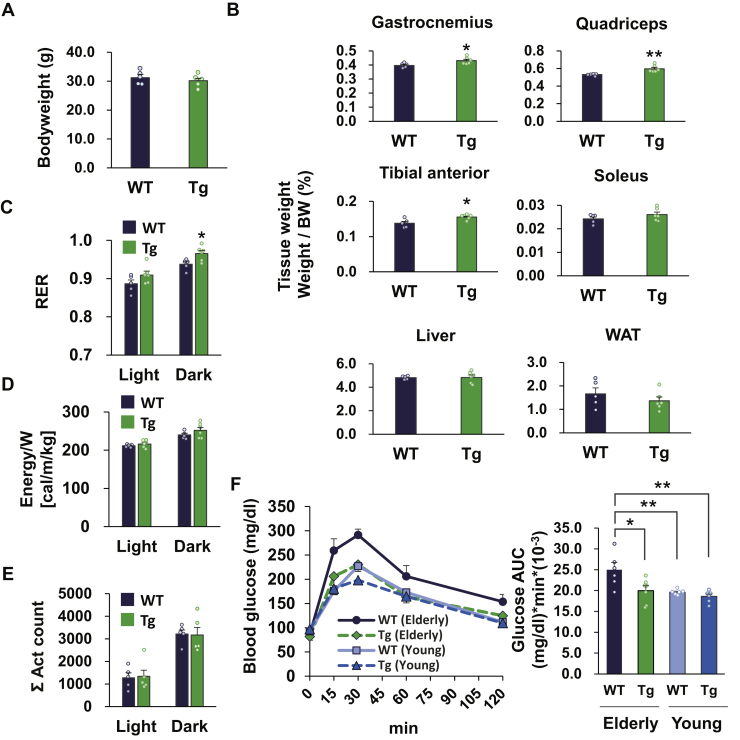
**Muscle TGR5 improves age-associated dysfunction of glucose homeostasis**. *A, B*, body weight (*A*) and tissue weight (*B*) of 23- to 24-month-old WT and Tg mice (n = 5–6). *C-E*, RER, energy expenditure, and act count of old WT and Tg mice were monitored for 48 h (n = 5–6). *F*, intraperitoneal glucose tolerance test in elderly (23- to 24-month-old) and young (2- to 5-month-old) WT and Tg mice (n = 6–7). The right panel shows the AUC. Data are mean ± S.E. Statistical analyses were conducted using a two-tailed unpaired Student’s *t* test or one-way ANOVA (Tukey–Kramer post hoc test). ∗, *p* < 0.05; ∗∗, *p* < 0.01. AUC, area under the curve; RER, respiratory exchange ratio.

Because aging is believed to be associated with glucose intolerance (15, 16), we determined the effect of muscle TGR5 on glucose clearance in aged mice. When WT mice and Tg littermates were compared at age 23 to 24 months, no significant difference was found in body weight, liver weight, and WAT weight, but there was a significant increase in skeletal muscle weight as well as at younger ages (Fig. 7, *A* and *B*). Next, we measured the RER, energy expenditure, and physical activity in aged WT and Tg mice and observed that skeletal muscle TGR5 increased the RER without affecting energy expenditure or physical activity (Fig. 7, *C*–*E*). These results indicate that TGR5 activation in skeletal muscle enhances glucose utilization in both older and younger mice. Finally, we performed an intraperitoneal glucose tolerance test (IPGTT) in elderly (aged 23–24 months) and young (aged 2–5 months) WT and Tg mice. Compared with young WT mice, elderly WT mice exhibited poor glucose clearance, whereas elderly Tg mice exhibited reduced blood glucose levels at various time points compared with elderly WT mice (Fig. 7*F*). The glucose area under the curve of elderly Tg mice was significantly lower than that of elderly WT mice, and the area under the curve level was comparable between young WT mice and elderly Tg mice, indicating that muscle TGR5 activation ameliorated age-associated glucose intolerance (Fig. 7*F*).

## Discussion

Various TGR5 agonists have been developed till date, which exert antiobesity and antidiabetic effects by activating thermogenesis in WAT and BAT and promoting GLP-1 secretion from enteroendocrine L cells (4, 5, 6, 7). It has been reported that the administration of TGR5 agonists, such as INT777, betulinic acid, and nomilin, to HFD-induced obese mice decreased body weight gain and blood glucose levels (10, 11, 12). We have also have previously demonstrated that TGR5 activation in skeletal muscle induces muscle hypertrophy (9). Skeletal muscle–specific TGR5 Tg mice exhibited increased muscle mass and enhanced muscle strength at both young and old ages. In general, the skeletal muscle is a major organ that consumes glucose, and hence, an increase in skeletal muscle mass is expected to improve glucose metabolism. However, the effects of skeletal muscle TGR5 on glucose and lipid metabolism remain unclear.

We first explored the role of muscle TGR5 in energy metabolism using skeletal muscle–specific TGR5 Tg mice that exhibited a characteristic increase in muscle mass (Fig. S1*A*). The exhaled breath analysis under the ND condition showed an increase in RER in Tg mice compared with control WT mice, indicating that TGR5 activation in skeletal muscle promotes glucose utilization (Fig. 1, *C* and *G*). As an increase in RER was not observed upon HFD challenge, it is possible that the utilization of glucose was promoted only when there was abundant dietary glucose available in the diet (Fig. 3, *C* and G). In contrast, there was no significant difference in energy expenditure between WT and Tg mice under both ND and HFD conditions (Fig. 1, D, H, and 3, D and H). In fact, body weight and food intake of Tg mice were comparable with those of WT mice under HFD challenge (Fig. 5, *A* and *B*). These data indicate that TGR5 does not promote energy expenditure and has no effect in preventing obesity at least in the mouse skeletal muscle. However, in the human skeletal muscle, it has been reported that TGR5 activation increases the expression of genes involved in energy production (4). Therefore, the effect of muscle TGR5 on energy metabolism may be different between mice and humans.

In glycolysis, which is one of the core metabolic pathways for energy production, glucose is converted into pyruvate by a 10-step enzymatic reaction. PFK is the most important rate-limiting glycolytic enzyme that catalyzes the conversion of F6P and ATP into F1,6BP, and ADP. PFK is phosphorylated by PKA, thereby modifying its enzymatic activity (17). A previous study demonstrated that activation of the cAMP-PKA pathway by epinephrine increases PFK activity through the stabilization of its tetrameric conformation in rabbit skeletal muscle (18). Moreover, it has been reported that PKA lowers the inhibition against PFK activity by lactate (19). These reports clearly indicate that PKA upregulates PFK activity in the skeletal muscle. Because TGR5 is known to activate PKA by increasing the intracellular cAMP levels, it is possible that PFK is activated in the skeletal muscle of Tg mice. To explore this possibility, we conducted metabolite profiling analysis by CE-TOF MS and observed a decrease in the levels of G6P and F6P, indicating the activation of PFK in the skeletal muscle of Tg mice (Fig. 2). In addition to PFK, there are two other known rate-limiting enzymes in glycolysis; one is hexokinase, which catalyzes the phosphorylation of glucose by ATP to G6P, and the other is pyruvate kinase, which catalyzes the transphosphorylation from PEP to ADP. The significant changes in the levels of G6P and F6P as well as 3PG and 2PG may partially be due to these rate-limiting steps other than PFK activation. These results obtained from the metabolite profiling analysis clearly explain the increased RER observed in Tg mice. Glycolysis in the skeletal muscle begins with glucose uptake from the blood or intracellular glycogen. The lack of difference in glycogen levels in the skeletal muscle between WT and Tg mice strongly suggests that the increased glycolysis in Tg mice was due to the increased uptake of blood glucose (Fig. S1*C*). Therefore, we conclude that activation of glycolysis in Tg mice is one of the reasons for improved glucose clearance.

As the skeletal muscle is a prominent organ of glucose disposal, TGR5-induced muscle hypertrophy and enhanced glucose utilization may lead to an increase in glucose consumption and improve hyperglycemia. To test this assumption, OGTT and ITT were performed using glucose-intolerant mice challenged by long-term HFD feeding. We observed that HFD-fed Tg mice exhibited improved glucose clearance compared with control WT mice (Fig. 5, *D* and *E*). In general, glucose intolerance in obese mice is caused by insulin resistance in peripheral tissues (20). In particular, the skeletal muscle has a significant influence on glucose clearance because it plays a major role in glucose uptake during hyperinsulinemia (21). Long-term HFD feeding promotes the accumulation of TG and lipid intermediates, such as diacylglycerol and ceramide, which hamper insulin signaling in the skeletal muscle (22, 23, 24, 25). Therefore, the TG content in the skeletal muscle correlates with insulin resistance (26, 27). Interestingly, we found little difference in the expression of genes related to lipid metabolism between the skeletal muscle of WT and Tg mice (Fig. S1*B*). Similar results were also obtained from TGR5-activated C2C12 myotubes, and as anticipated, TGR5 activation had no influence on intramuscular TG accumulation under palmitate treatment (Fig. 4, *A* and *B*). Consequently, TGR5 activation did not improve palmitate-induced insulin resistance in C2C12 myotubes, as well as HFD-induced insulin resistance in mice (Fig. 6, *A* and *B*). Furthermore, the levels of fasting plasma glucose, TG, NEFAs, and insulin, which are indicators of insulin resistance, were not different between Tg and WT mice after HFD challenge (Fig. S3). These results are consistent with our data showing that increased glucose utilization in Tg mice occurs when glucose is abundantly ingested in the form of ND rather than HFD. Our findings suggest that the improvement of glucose clearance by muscle TGR5 is independent of enhanced insulin sensitivity but rather caused by skeletal muscle hypertrophy and increased glucose utilization.

Unlike our study results, a recent study reported that TGR5 activation in the skeletal muscle improves insulin sensitivity and glucose homeostasis in a diabetic mice model (28). In the present study, palmitate-induced insulin resistance was prevented by TGR5 ligand and it was canceled by TGR5 silencing. Although the reason for this discrepancy remains unclear, it might be due to the type of the agonist used, as various agonists can lead to a bias in downstream signaling of G protein–coupled receptors (29).

In addition to obesity, aging is an important risk factor for glucose intolerance and insulin resistance (15, 16). Therefore, we examined the effects of skeletal muscle TGR5 on glucose metabolism in aged mice and detected better glucose tolerance in aged Tg mice (Fig. 7*F*). In general, age-related glucose intolerance is caused by body fat accumulation (30, 31), physical inactivity (30), and skeletal muscle atrophy (32). We compared body weight, fat weight, and physical activity in elderly WT and Tg mice and found that these parameters were comparable (Fig. 7, A, B and E). In contrast, there were significant increases in skeletal muscle weight and RER in elderly Tg mice (Fig. 7, *B*–*C*). These results suggest the involvement of muscle hypertrophy and increased glucose utilization in the improvement of glucose clearance in both elderly mice and HFD-induced obese mice.

**Figure 8:**
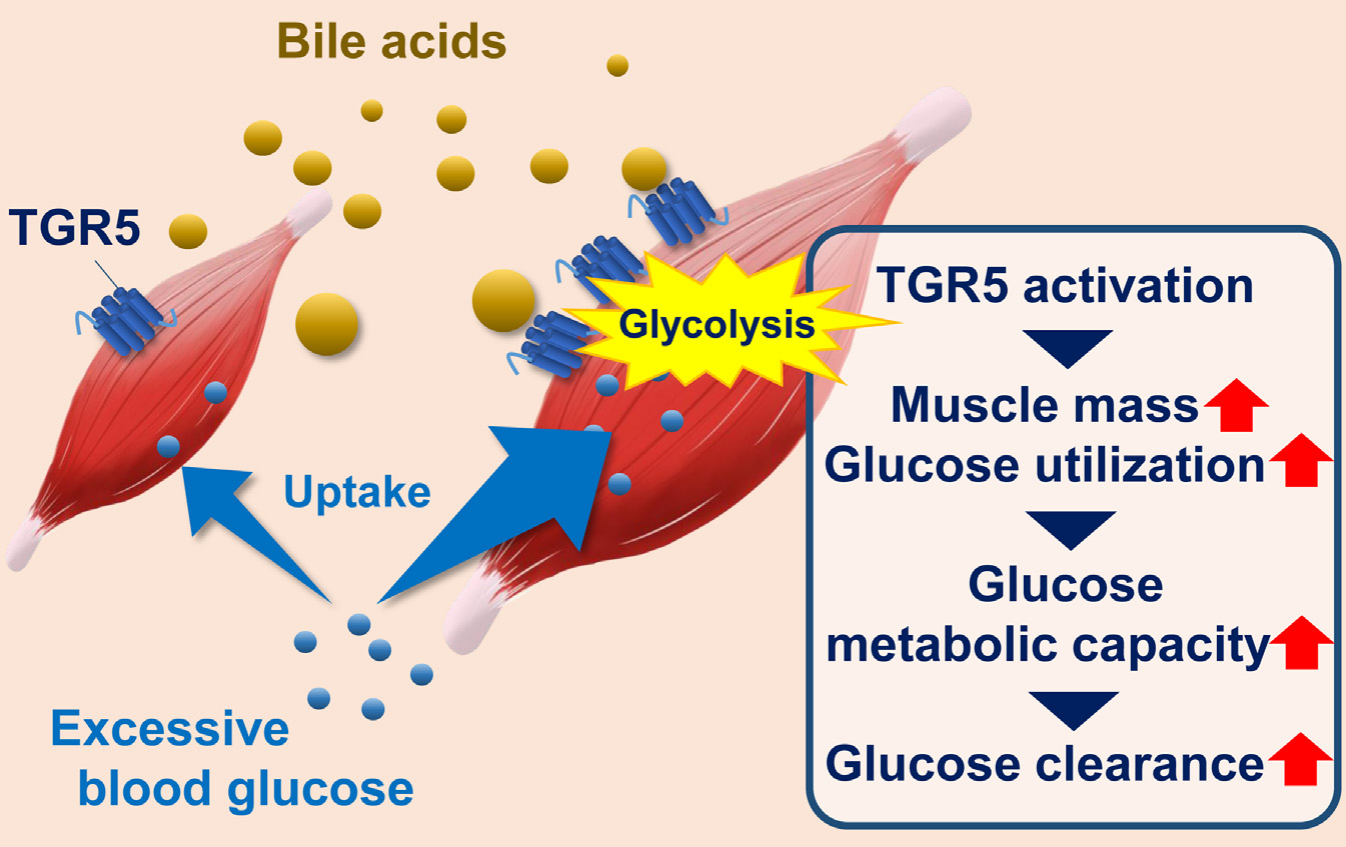
**Schematic representation of the effect of muscle TGR5 on glucose clearance**.

In conclusion, our study findings reveal that muscle TGR5 improves glucose intolerance induced by HFD or aging without affecting muscle insulin sensitivity. The results of a series of experiments, including exhaled breath analysis and metabolite profiling, suggest that TGR5-induced skeletal muscle hypertrophy and increased glucose utilization through the activation of glycolytic flux contribute to improved glucose clearance (as summarized in the schema in Fig. 8). Considering that we and others have discovered various TGR5 agonists from food components till date (12, 33, 34, 35), it will be of great interest to investigate whether dietary modifications by these food components could improve glucose intolerance through muscle TGR5 activation in mice and humans.

## Experimental procedures

### Antibodies

The following antibodies were used in this study: anti-Akt (#9272) and anti-phospho-AKT (thr308) (#9275) (Cell Signaling Technology); anti-FLAG (M2) and anti-β-actin (AC-15) (Sigma); anti-GAPDH (10494-1-AP) (Proteintech); and horseradish peroxidase–coupled anti-mouse IgG and anti-rabbit IgG (Jackson Immune Research).

### Animals and diets

Muscle-specific TGR5 transgenic mice were generated as previously described (9). In brief, 3 × FLAG hTGR5 was cloned into MCK promoter–containing plasmid, and the purified transgene was injected into C57BL/6 oocytes. Mice were housed with a 12:12-h light–dark cycle and provided free access to water and standard chow (Labo MR Stock; Nosan Corporation Bio-Department). HFD pellets with 60% energy supplied by fat were purchased from Research Diet (D12492). The number of mice used in each experiment is described in the figure legend. All animal experiments were conducted according to the guidelines of the Animal Usage Committee of the University of Tokyo.

### Metabolic analysis

O_2_ consumption and CO_2_ production in Tg and WT mice were measured using an ARCO-2000 Mass Spectrometer (ARCO system) with one mouse per chamber as previously described (36). The chambers were maintained at 21 °C ± 3 °C, with 50% ± 10% relative humidity. The physical activity was quantitated using an infrared beam sensor (NS-AS01, Neuroscience) placed approximately 11 cm above the center of the cage.

For OGTT, mice were fasted for 16 h, and glucose water was orally administered (2 mg/g body weight). For IPGTT, mice were fasted for 16 h, and glucose water was intraperitoneally administered (1 mg/g body weight). For ITT, mice were fasted for 6 h and then injected with insulin intraperitoneally (0.75 IU/kg body weight). Blood glucose level was measured using a handheld glucometer (Ascensia Breeze 2; Bayer Diagnostics).

### Serum biochemistry

Levels of serum glucose, TG, and NEFAs were determined using kits purchased from FUJIFILM Wako Pure Chemical. Serum insulin concentrations were estimated using the Mouse Insulin ELISA Kit purchased from FUJIFILM Wako Shibayagi Corporation.

### Metabolite profiling with CE-TOF MS

Gastrocnemius muscles collected from WT and Tg mice were homogenized and used for the analysis of ionic metabolites. Before analysis, hydrophobic and high-molecular-weight compounds were removed by the preparative processes of liquid–liquid separation using chloroform and water and ultrafiltration using a 5-kDa cutoff filter (37). A comprehensive analysis of ionic metabolites by CE-TOF MS was performed as described previously (38).

### Cell culture

C2C12 myoblasts obtained from ATCC were cultured in Dulbecco’s modified Eagle’s medium (DMEM) supplemented with 10% fetal bovine serum, 100 U/ml penicillin, and 100 μg/ml streptomycin. To induce differentiation, C2C12 myoblasts were cultured in DMEM supplemented with 2% horse serum, 100 U/ml penicillin, and 100 μg/ml streptomycin for 4 to 5 days. Cells were maintained at 37 °C in 95% humidity with 5% CO_2_.

For TGR5 overexpression experiments, C2C12 myotubes were infected overnight with 2.5 × 10^6^ plaque-forming units/ml adenovirus medium. Then, the cells were washed with PBS three times and incubated with fresh medium.

### Real-time PCR

Total RNA was extracted from C2C12 myotubes or the skeletal muscle of mice using ISOGEN (NIPPON GENE), according to the manufacturer’s instructions. The high-capacity cDNA reverse transcription kit (Applied Biosystems) was used to synthesize and amplify cDNA from total RNA. Quantitative real-time PCR analyses were performed using an Applied Biosystems StepOnePlus instrument. Expression was normalized to an 18S ribosomal RNA (18S). The primers used for the PCR analysis are described in Supporting information Table S1.

### Immunoblotting

Cells and mouse skeletal muscle were lysed in radio immunoprecipitation assay buffer (50 mM Tris-HCl (pH 8.0), 150 mM NaCl, 1% (v/v) Triton X-100, 0.5% (w/v) deoxycholate, and 0.1% (w/v) SDS) supplemented with a protease inhibitor mixture (Nacalai Tesque) and a phosphatase inhibitor mixture (Sigma-Aldrich). The lysates were subjected to SDS-PAGE, transferred to a polyvinylidene difluoride membrane (Millipore, Billerica, MA), and then probed with the antibodies indicated in the figure legends. For cell lysates of C2C12 myotubes, the β-actin protein was used as an internal control, and for mouse skeletal muscle lysates, GAPDH protein was used as a control due to the low expression of β-actin *in vivo*.

### Intracellular TG measurements

C2C12 myotubes were washed with PBS, and lipids were extracted by hexane in 2-propanol (3:2, v/v). The levels of intracellular TG were determined using the Triglyceride E-test Wako Kit (FUJIFILM Wako Pure Chemical) and normalized to the levels of total cellular protein determined using a BCA protein assay kit (Pierce), according to each manufacturer’s instructions.

### Statistical analysis

All data are presented as mean ± SE. Two-tailed unpaired Student’s *t* tests or one-way ANOVAs (Tukey–Kramer post hoc test) were used to determine *p* values. Statistical significance was defined as *p* < 0.05.

## Data availability

All the data are in the manuscript.

## Conflict of interest

The authors declare that they have no conflicts of interest with the contents of this article.
