# Supplementary material for: Muscle-specific TGR5 overexpression improves glucose clearance in glucose-intolerant mice
Source: J Biol Chem. 2020 Dec 4;296:100131. doi: 10.1074/jbc.RA120.016203 (PMC7949087; doi:10.1074/jbc.RA120.016203)
Supplement: Tables and figures [file mmc1.pdf]

Supporting Information

Muscle-specific TGR5 overexpression improves glucose clearance in glucose-intolerant mice

Takashi Sasaki[1*], Yuichi Watanabe[1], Ayane Kuboyama[1], Akira Oikawa[2,3], Makoto Shimizu[4], Yoshio Yamauchi[1], Ryuichiro Sato[1, 4, 5, *]

[1] Food Biochemistry Laboratory, Department of Applied Biological Chemistry, Graduate School of Agricultural and Life Sciences, University of Tokyo, Tokyo 113-8657, Japan
[2] RIKEN Center for Sustainable Resource Science, Yokohama, Kanagawa 230-0045, Japan
[3] Faculty of Agriculture, Yamagata University, Tsuruoka, Yamagata 997-8555, Japan
[4] Nutri-Life Science Laboratory, Department of Applied Biological Chemistry, Graduate School of Agricultural and Life Sciences, University of Tokyo, Tokyo 113-8657, Japan
[5] AMED-CREST, Japan Agency for Medical Research and Development, Tokyo 100-0004, Japan

Table S1. List of Real-time PCR primers

Gene name	Forward	Reverse
**Cd36**	CTTCCACATTTCCTACATGCAA	ATCCAGTTATGGGTTCCACATC
**Cpt-1b**	ACCGTGAAGAGATCAAGCCGGT	TCTCTTTGCCTGGGATGCGTGT
**CytC**	ACAAGAAGACTCAAATGTGTTTCAGTTT	TGCACTGTCAAGAATAGACAGTTGC
**Dgat1**	GTGCACAAGTGGTGCATCAG	CAGTGGGACCTGAGCCATCA
**Dgat2**	CAGCAAGAAGTTTCCTGGCAT	CCTCCCACCACGATGATGAT
**Glut4**	GAGCTGAAGGATGAGAAACGGA	CATTGATGCCTGAGAGCTGTTG
**Lpl**	CTTCTTGATTTACACGGAGGT	ATGGCATTTCACAAACACTG
**Nr4a3**	TCAGCCTTTTTGGAGCTGTT	TGAAGTCGATGCAGGACAAG
**Pdk4**	AAAGGACAGGATGGAAGGAATCA	TTTTCCTCTGGGTTTGCACAT
**Pgc-1a**	TTCTGGGTGGATTGAAGTGGTG	TGTCAGTGCATCAAATGAGGGC
**Ppara**	CTCGCGTGTGATAAAGC	CGATGCTGTCCTCCTTG
**Pparg**	GGACTGTGTGACAGACAAGATTTG	CTGAATATCAGTGGTTCACCGC
**Ppard**	GCCTCGGGCTTCCACTAC	AGATCCGATCGCACTTCTCA
**Srebp-1c**	GAGCCATGGATTGCACATTT	CGGGAAGTCACTGTCTTGGT
**hTGR5**	TTACCTGGAGGCAGGCAAGG	CTGGCACTTCCTAGGGAGAG
**18S**	ACCGCAGCTAGGAATAATGGA	GCCTCAGTTCCGAAAACCA

Figure S1

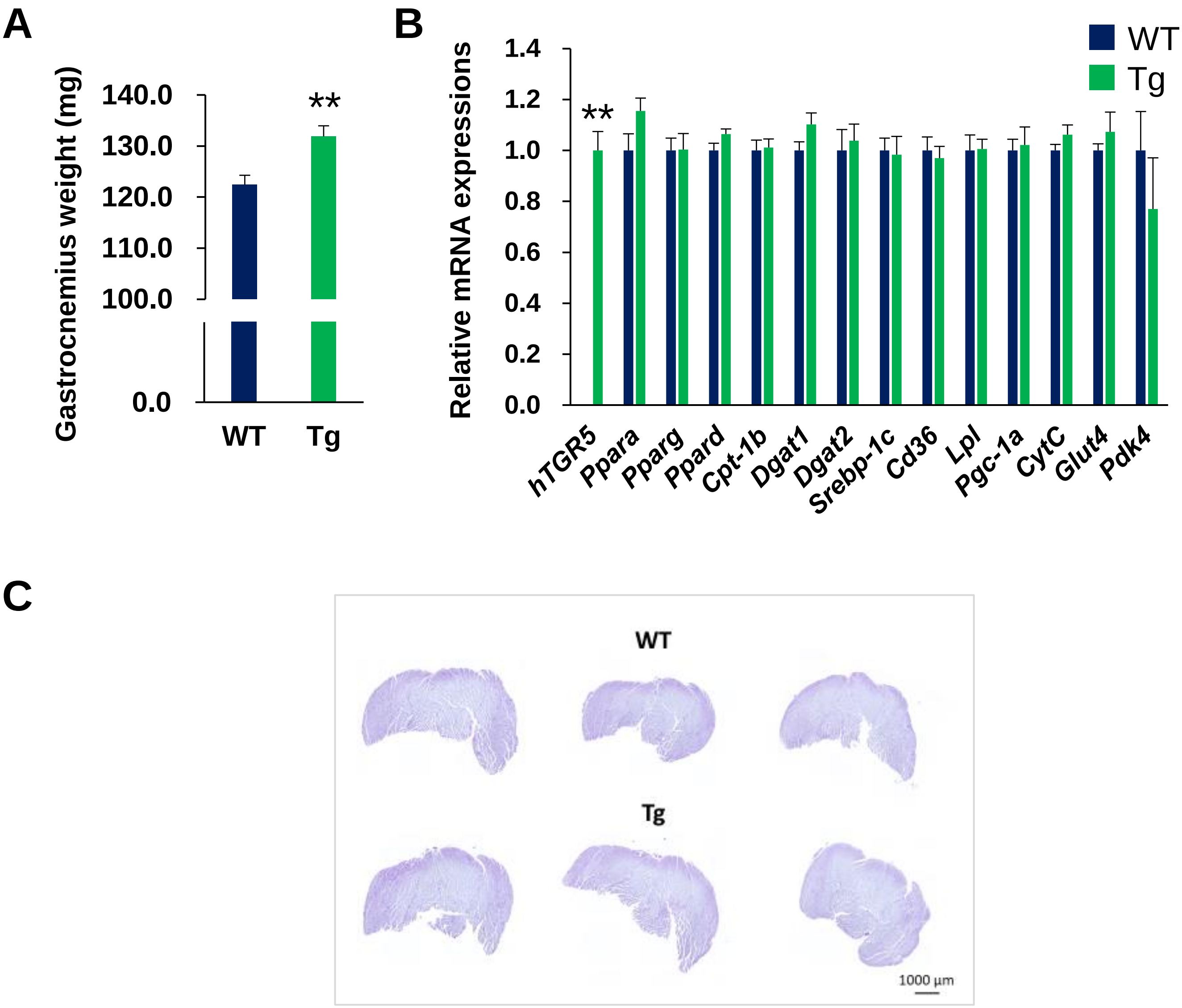

Figure S1. Effects of TGR5 on muscle mass and glucose and lipid metabolism-related genes expression in mice. (A) Gastrocnemius weight of 8-week-old male littermates of the WT and Tg mice (n = 8–9). (B) The gastrocnemius was isolated and the mRNA levels of glucose and lipid metabolism-related genes were measured (n = 8–9). (C) PAS staining in gastrocnemius of WT and Tg mice. Data are means ± S.E. Statistical analyses were conducted using a two-tailed unpaired Student's *t*-test. **, $p < 0.01$.

Figure S2

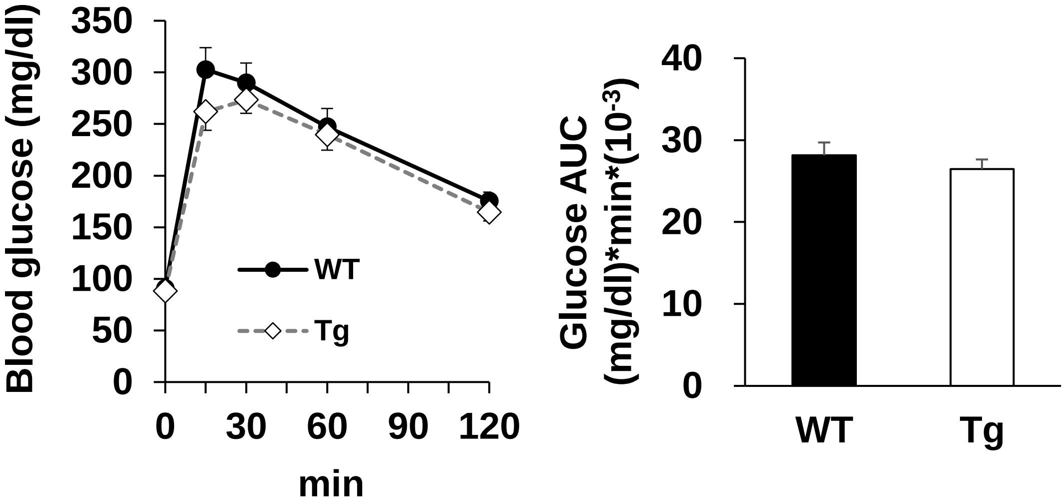

Figure S2. Blood glucose level during OGTT in WT and Tg mice. OGTT in 8-week-old WT and Tg mice fed ND. The right panel shows AUC. Data are mean ± S.E. (n = 8–10).

Figure S3

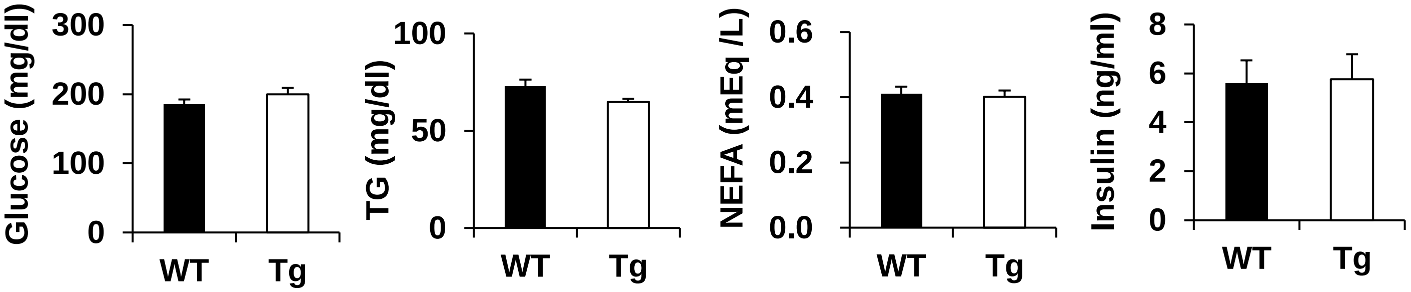

Figure S3. fasting blood glucose, TG, NEFA, and Insulin in HFD-fed Tg mice. Blood glucose, TG, NEFA, and insulin levels in WT and Tg mice after 15 weeks of HFD feeding followed by 4 h fasting. Data are means ± S.E. (n = 12–13).

Figure S4

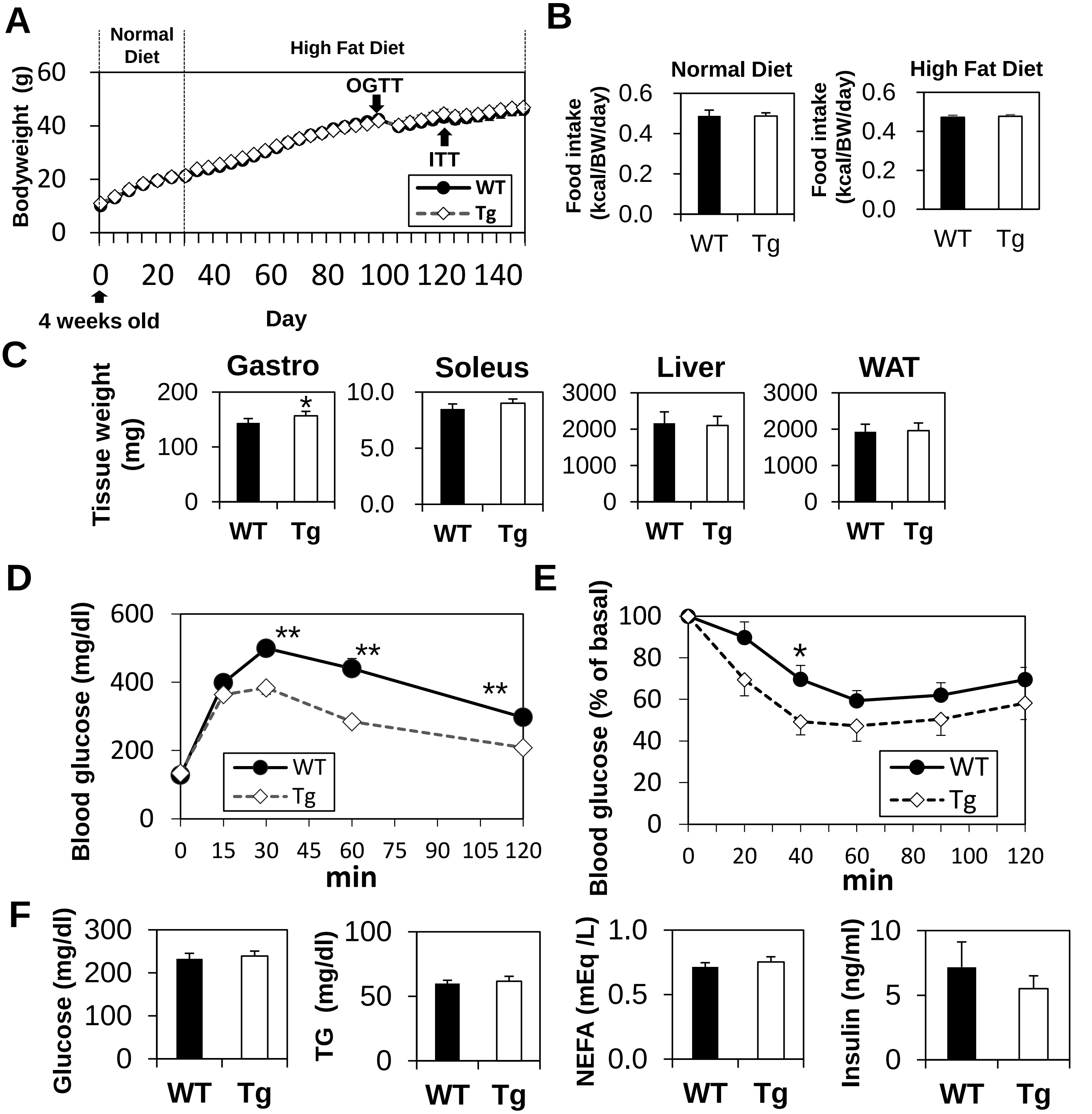

Figure S4. Muscle TGR5 prevents obesity-induced dysfunction of glucose homeostasis (Line B). Analysis of HFD-fed Tg mice (Line B) and WT littermates (n = 8–10). (A-C) Bodyweight (A), food intake (B), and tissue weight (C) of WT and Tg mice fed an HFD. (D, E) Glucose tolerance test (D) and insulin tolerance test (E). (F) Blood glucose, TG, NEFA, and insulin levels in WT or Tg mice after 16 weeks of HFD feeding followed by 4 h fasting. Data are means ± S.E. Statistical analyses were conducted using one-way ANOVA (Tukey–Kramer post hoc test).* $p < 0.05$, ** $p < 0.01$
